# Characterizing Fishing Effort and Spatial Extent of Coastal Fisheries

**DOI:** 10.1371/journal.pone.0014451

**Published:** 2010-12-29

**Authors:** Kelly R. Stewart, Rebecca L. Lewison, Daniel C. Dunn, Rhema H. Bjorkland, Shaleyla Kelez, Patrick N. Halpin, Larry B. Crowder

**Affiliations:** 1 Protected Resources Division, National Marine Fisheries Service, Southwest Fisheries Science Center, La Jolla, California, United States of America; 2 Biology Department, San Diego State University, San Diego, California, United States of America; 3 Marine Geospatial Ecology Lab, Duke University Marine Laboratory, Nicholas School of the Environment, Duke University, Beaufort, North Carolina, United States of America; 4 Duke Center for Marine Conservation, Duke University Marine Laboratory, Nicholas School of the Environment, Duke University, Beaufort, North Carolina, United States of America; University of Glamorgan, United Kingdom

## Abstract

Biodiverse coastal zones are often areas of intense fishing pressure due to the high relative density of fishing capacity in these nearshore regions. Although overcapacity is one of the central challenges to fisheries sustainability in coastal zones, accurate estimates of fishing pressure in coastal zones are limited, hampering the assessment of the direct and collateral impacts (e.g., habitat degradation, bycatch) of fishing. We compiled a comprehensive database of fishing effort metrics and the corresponding spatial limits of fisheries and used a spatial analysis program (FEET) to map fishing effort density (measured as boat-meters per km^2^) in the coastal zones of six ocean regions. We also considered the utility of a number of socioeconomic variables as indicators of fishing pressure at the national level; fishing density increased as a function of population size and decreased as a function of coastline length. Our mapping exercise points to intra and interregional ‘hotspots’ of coastal fishing pressure. The significant and intuitive relationships we found between fishing density and population size and coastline length may help with coarse regional characterizations of fishing pressure. However, spatially-delimited fishing effort data are needed to accurately map fishing hotspots, i.e., areas of intense fishing activity. We suggest that estimates of fishing effort, not just target catch or yield, serve as a necessary measure of fishing activity, which is a key link to evaluating sustainability and environmental impacts of coastal fisheries.

## Introduction

Coastal and nearshore ecosystems are some of the richest areas of marine biodiversity globally [1]. Coastal regions also support considerable human populations; at least 50% of people on Earth live and work within 200 km of a coast [2]. Coastal zones are threatened by many factors – pollution, habitat loss and degradation, intense harvest of marine resources – that are driven by human activities on land and at sea [3], [4]. As human populations have expanded, fishing pressure in coastal areas has intensified and coastal fisheries increasingly play a central role in the economies and livelihoods of people around the world [5], [6]. Coastal zones are home to a wide range of fishing fleets, from artisanal or small-scale vessels to large-scale industrial vessels that employ an equally broad range of fishing gears and practices. These fleets support millions of households and drive local, national and, in some areas, international markets and economies.

For over a decade, there has been growing concern regarding the sustainability of fisheries worldwide [7]–[10]. Evidence of declining catches, depleted or modified shorelines, and struggling coastal communities across the globe point to synergistic challenges of overexploitation, overcapacity, and lack of management [11], [12]. To date, much of the attention has been focused on quantifying fishery catches as a measure of fishing intensity in an area [4], [13]. While catch information is useful, it does not directly address one of the fundamental issues of fisheries sustainability, namely direct and collateral impacts by fishing gear on habitats, target and non-target species [14], [15]. While catches in many areas have fluctuated, coastal fishing effort (amount of gear deployed) has been increasing markedly in many fishing regions [11], [16]–[18]. The widespread pattern of variable catches associated with substantially higher gear deployment illustrates the limitations of only monitoring catch statistics: many of the collateral ecological impacts from fisheries i.e., habitat degradation and higher levels of discarded non-target catch [19], [20], are directly related to the amount of gear deployed rather than to the amount of target yield extracted.

One of the central challenges to understanding the impact of fisheries on coastal ecosystems is the lack of fishing effort data (i.e., the number of boats, the amount of gear deployed, or the frequency of fishing activities). Mapping coastal fishing effort is one approach to quantify the relative intensity of fishing pressure across large areas. Efforts to describe and map coastal fisheries have been hindered by the obstacles associated with measuring and describing fishing effort, particularly for small-scale or artisanal fleets [21]. These challenges include a lack of resources directed toward data collection, the distant and dispersed nature of the fisheries [22], [23], disparities among data sources, limited data availability in some areas, and a scarcity of spatial information. The changeable nature of coastal fisheries (i.e., target species, gear types) over short time scales also challenges characterizations of this fisheries sector [24]. Recent efforts to characterize coastal fishing pressure have highlighted the importance of quantifying fisheries activity as a first step to develop more sustainable fisheries management plans [24], [25].

Using a mapping tool developed to assess fishing effort in Caribbean fisheries [23], we integrated United Nations Food and Agriculture Organization (FAO) data, national fisheries reports and published research to characterize fishing pressure in the coastal waters of six large marine regions. Through this process we were able to map regional-scale patterns of coastal fishing effort and examine the relationship among average fishing effort densities, economic development levels and other demographic parameters within and among countries and regions. We present a novel approach to mapping fishing effort across disparate ocean regions, based on the development of a common fishing effort metric that allows for interregional comparisons. The goal of our analysis was to compare the relative density of coastal fishing effort and to consider potential socioeconomic and physical correlates of fishing density among six different marine regions. Our approach provides a method for quantifying fishing effort and may serve as a means of identifying areas where overcapacity may threaten fisheries sustainability and the integrity of coastal ecosystems.

## Methods

We focused our analyses on six regions: West Africa, the West Indian Ocean, Southeast Asia, the Eastern Tropical Pacific, the Caribbean, and the Southwest Atlantic. These regions were selected because they are reported to have high but poorly documented coastal fishing effort across varying fisheries sectors, e.g., artisanal to industrial [24], [26]–[28]. We defined coastal fisheries as those that deployed gear from shore out to either 50 km in distance or from shore to 200 m in depth [13].

### Mapping fishing effort

We initiated the development of our dataset by aggregating the FAO country profiles (http://www.fao.org/countryprofiles) for each of the six regions. Building on fishing effort information found in the country profiles, we then conducted a comprehensive review of all published and in-country papers and reports that contained fisheries data and were available to us. From these sources, we recorded all reported fishing effort variables: fishery name, type of gear used, the total number of boats, horsepower, boat size, length range of boats, amount of gear deployed per vessel, information on fishing season (trips per year, days fished per year, etc.), major ports, distance from shore (maximum and minimum), bottom depth where fishing occurred, target species, and fishing season duration. In general, we used information from the past five years, but older fisheries data were used if no other data were available. Older fisheries data may be relevant to more current analyses, particularly when there is no information about known fishing activity [6]. A complete list of data sources is provided in Appendix S1 (Supporting Information). We included all types of fishing gear in our database.

We extracted three basic metrics that were most commonly reported across regions: the number of boats, the length of boats, and the spatial boundary of the fishery. From these parameters, we calculated the amount of fishing effort for each fishery as the product of number of boats and boat length to yield boat-meters [23]. Boat length and number, along with other fisheries characteristics have been shown to be among the key variables used to describe fishing activity [29]–[31]. Based on our literature search and research, we found that engine-size parameters were less documented and more highly variable within regions than boat lengths and boat numbers. For fisheries where boat length was not reported, we used the average length of vessel reported within a gear for a corresponding development level (i.e., artisanal vs. industrial) in the country or region. We retained the range of values reported in the literature as these values contributed to the standard deviations included in Tables 1 & S1.

**Table 1:** Average densities (boat-meters/km^2^) along with standard deviation and sample size (number of fisheries) by fishing gear across six marine regions.

Fishing gear	WA	WIO	SEA	ETP	CAR	SWA	Total
Beach seine	na	na	0.04 (1)	na	3.18±4.76 (8)	na	2.83±4.57 (9)
Diving	na	na	na	0.91 (1)	0.2±0.30 (7)	na	0.29±0.37 (8)
Driftnet	0.01 (1)	na	na	na	na	na	0.01 (1)
Gillnet	2.78±1.8 (2)	1.9±3.10 (8)	1.34±0.93 (3)	0.02±0.00 (2)	1.77±2.73 (10)	0.04±0.03 (2)	1.58±2.43 (27)
Hook and line	0±0.00 (3)	0.21±0.14 (3)	0.33±0.08 (2)	0.14 (1)	0.24±0.66 (13)	na	0.2±0.51 (22)
Longline	0.14±0.34 (7)	0.03±0.04 (3)	na	0.35±1.24 (18)	4.97±16.68 (13)	0 (1)	1.71±9.33 (42)
Mixed gear	8.61±7.49 (22)	1.56±3.16 (27)	7.58±8.32 (34)	7.11±12.25 (13)	5.32±19.04 (23)	0.98±1.25 (3)	5.79±10.85 (123)
Purse seine	0.09±0.16 (9)	0 (1)	0.08±0.14 (4)	0.1±0.16 (8)	na	na	0.09±0.15 (22)
Trap	0.01 (1)	0.6±0.91 (4)	na	0.03 (1)	1.89±4.43 (17)	na	1.5±3.85 (23)
Trawl	0.18±0.43 (23)	1.03±2.18 (15)	0.43±0.67 (7)	0.39±0.76 (19)	0.22±0.37 (15)	0.05±0.04 (5)	0.4±1.06 (84)
Weir	na	0.74±0.51 (4)	na	na	na	na	0.74±0.51 (4)
Total Average Density	2.95±5.78 (68)	1.21±2.55 (65)	5.21±7.57 (51)	1.72±6.11 (63)	2.55±10.83 (106)	0.3±0.71 (11)	2.55±7.56 (364)

WA  =  West Africa, WIO  =  West Indian Ocean, SEA  =  Southeast Asia, ETP  =  Eastern Tropical Pacific, CAR  =  Caribbean, and SWA  =  Southwest Atlantic.

na  =  no fisheries designated as using this type of gear.

Data were then input into a spatial analysis program, the Fishing Effort Envelope Tool (FEET) [23]. FEET combines information on distance from shore, distance from port, and the depth of the fishery to delimit the potential area in which a fishery may operate. Using various algorithms (Appendix S1, Supporting Information), FEET then distributes fishing effort across the fishing effort envelope in 1 km^2^ cells. In this study we created fishing effort envelopes for all known fisheries (n = 364) from 96 countries within six regions. While this is likely to be a substantial underestimate of the actual number of fisheries that exist across these regions, these were the fisheries for which we had the necessary information for this mapping exercise. All information regarding the location or spatial extent of each fishery was used to map fishing effort. When explicit information on the location of the fishery was not reported, we estimated the model parameters (depth and distance from shore) based on the known distribution of target species, the general characteristics of associated features, or the fishing gear used. The mapped data includes three broad fishing gear categories: gillnets, longlines and trawls. These three gear categories, which include different sub-types within each category (e.g., bottom trawls and mid-water trawls within ‘trawls’), are recognized by the FAO as major fishing gears (http://www.fao.org/fishery/topic/1617/en) and provide a means of comparing fishing effort among disparate ocean regions. In addition, other gear types (seines, traps, etc.) were mapped and included in density estimates.

We distributed fishing effort in the coastal zone using an inverse distance-from-shore weighting, excluding high-sea fisheries. For industrial and super-industrial sectors, fishing effort was distributed evenly across the fishing effort envelopes. The fishing effort envelopes were restricted to the coastal zone and then fishing effort was summarized for each 1 km^2^ grid cell. Prior to map creation, we solicited input from experts (managers and biologists in the field), and their feedback was used to update the fishing effort tables and resulting maps. Fishing effort was calculated as boat-meters divided by the spatial extent (in km^2^) of the fishing area. This effort metric created a density value (boat-meters/km^2^) that was used throughout the six regions. For additional details on metric development, see Appendix S1 (Supporting Information). Using the common metric of fishing density, we tested for significant patterns in density by gear or overall density across the six regions. For interregional comparisons of fishing densities, we log-transformed fishing densities and used one-way ANOVA to test for differences. We tested variables for homogeneity of variance using Bartlett's test [32].

Data limitations precluded detailed error estimation. However, in addition to retaining data ranges wherever possible to generate standard deviations around our estimated values, we also calculated our fishing density estimates in two ways because of the influence that spatial data has on fishing density, i.e., fishing effort that is associated with high-resolution spatial data will always yield a higher density estimate than effort that has limited spatial information. Using the results generated by FEET, we calculated average fishing densities for each country under two different scenarios of fishing effort distribution. In the first scenario, we assumed fishing effort was distributed throughout the entire coastal zone (CMD). In the second scenario, we distributed fishing effort only in the estimated fished cells (FMD). Fished cells (FMD) are defined as those areas that intersected one or more fishing effort envelopes as generated by FEET. We report both values to highlight the influence of spatial information accuracy on our results. Data limitations also precluded any formal consideration of temporal variability in fishing densities. Thus, our results characterize annual fishing pressure but do not capture the monthly or seasonal changes that undoubtedly occur.

### Correlates to fishing pressure

We analyzed mean fishing effort data for all countries to consider whether national socioeconomic or physical variables can serve as correlates or proxy variables for fishing pressure in an area. We considered the relationships between the fishing effort metrics and five country-level economic and physical variables: Human Development Index (HDI) [33], Gross Domestic Product (GDP), per capita GDP (PCGDP), population size and length of coastline. Data for the variables were obtained from the International Monetary Fund World Economic Outlook Database [34] and FAO country profiles. We considered the strength of the relationships between this group of variables and fishing density across all gears using generalized linear and general regression models in STATISTICA. In the generalized linear model, we allowed the model to be related to the response variable via a log link function and model selection was based on Akaike's information criterion (AIC). We also used a one-way ANOVA to test for the relationship between HDI and fishing density, independent of the continuous variables.

## Results

### Mapping fishing effort

**Figure 1:**
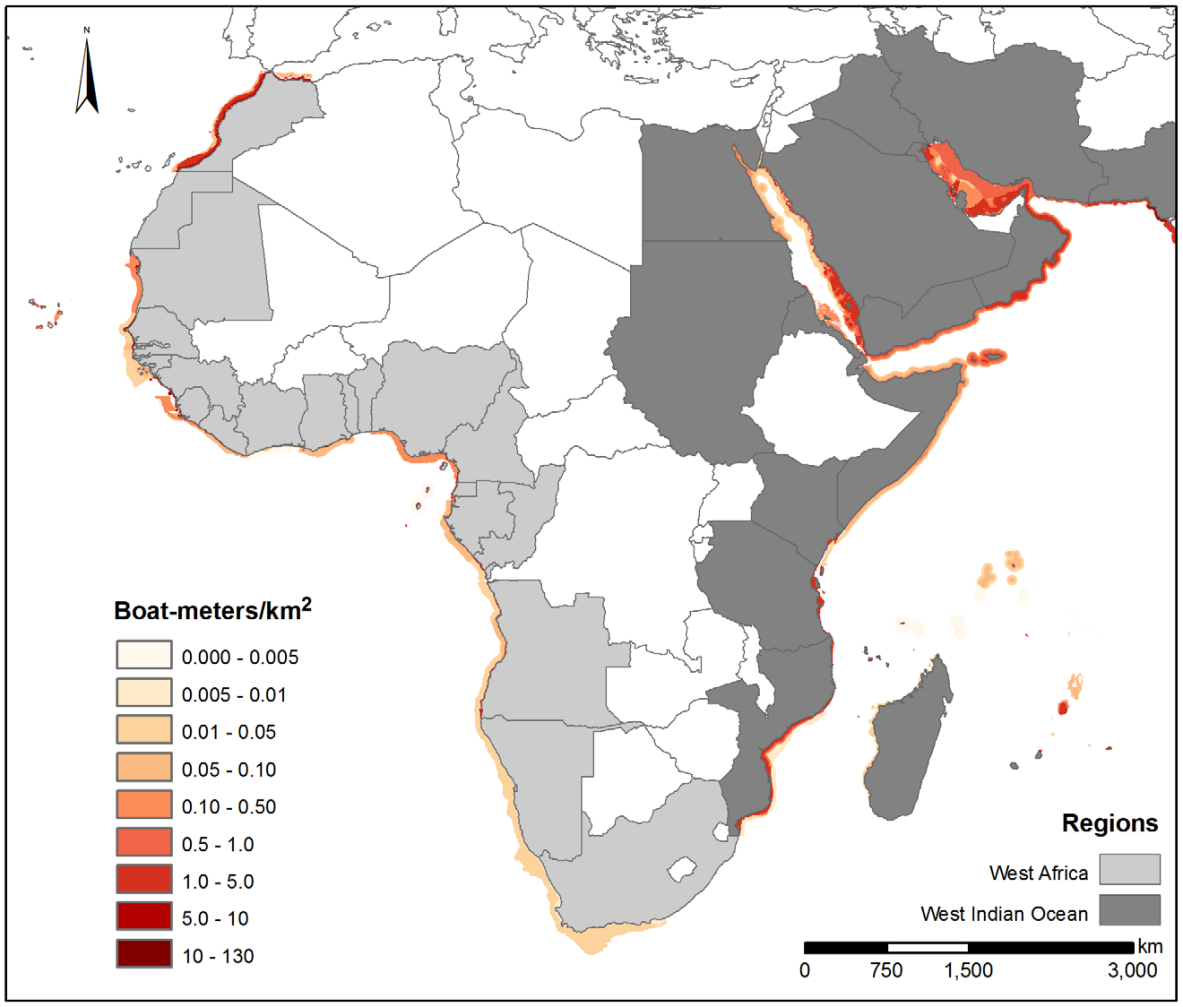
Fishing densities (boat-meters/km^2^) for West Africa and the West Indian Ocean.

**Figure 2:**
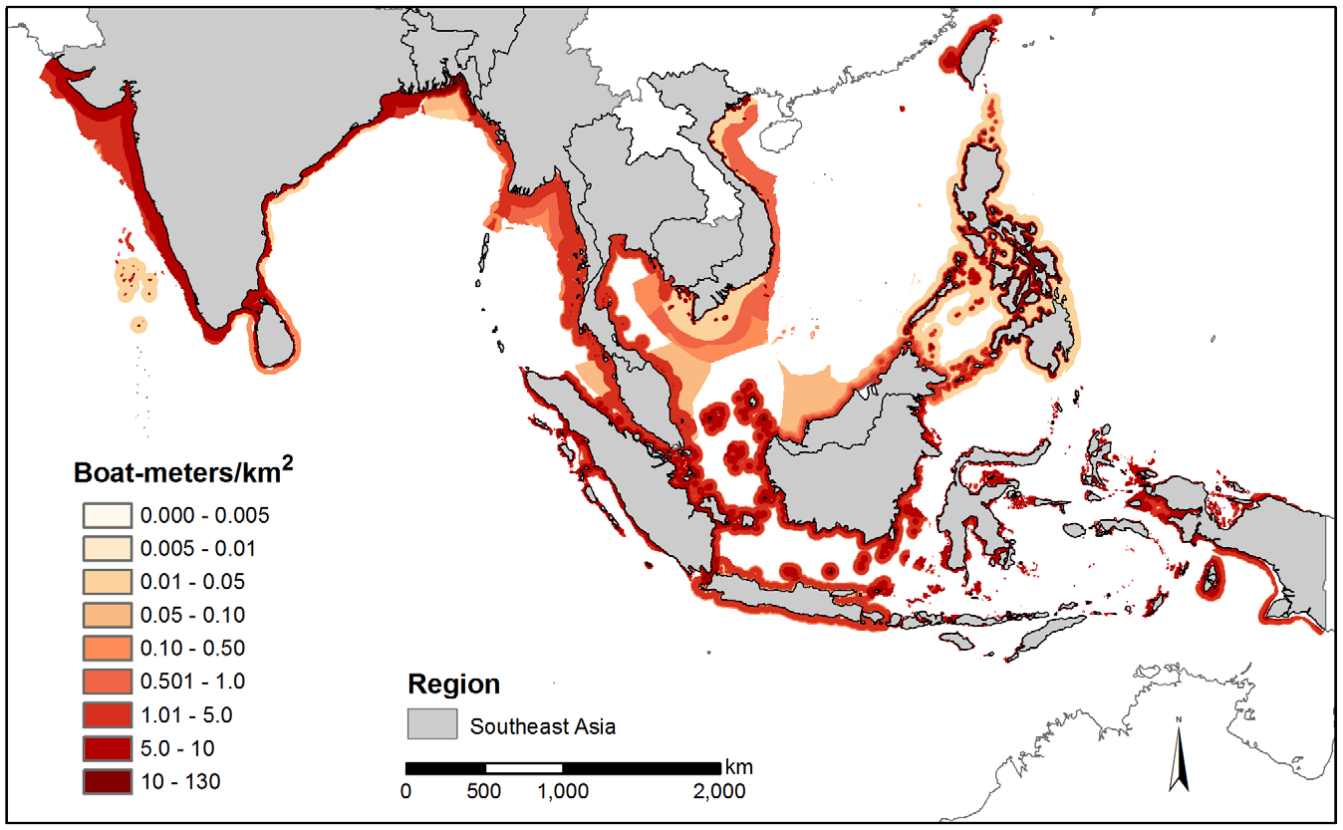
Fishing densities (boat-meters/km^2^) for Southeast Asia.

**Figure 3:**
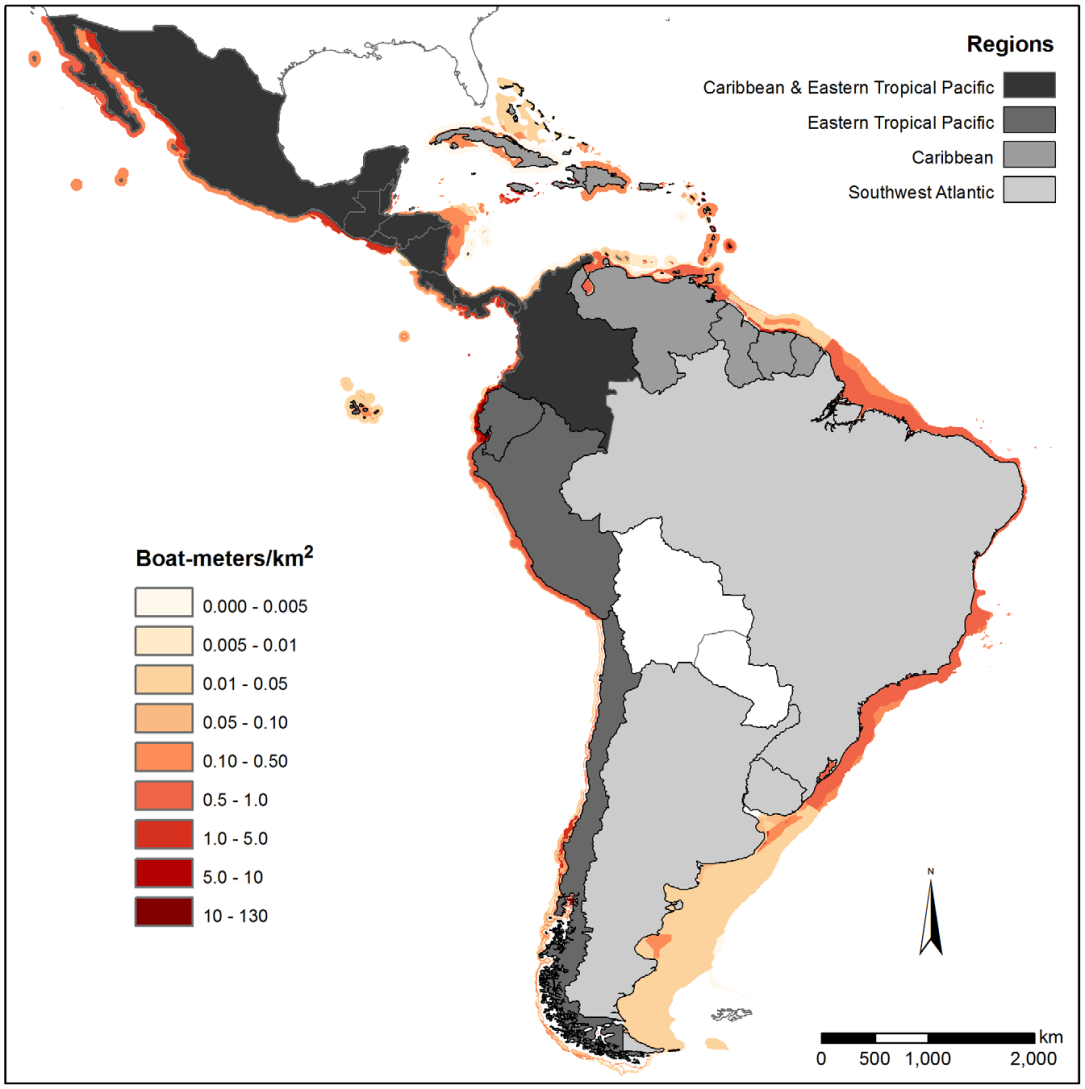
Fishing densities (boat-meters/km^2^) for the Eastern Tropical Pacific, Caribbean and Southwest Atlantic.

Although the entire coastal zone mean density calculation (CMD) for some countries yielded lower fishing densities, we characterized these values as underestimates of fishing density. Instead, we selected FMD because it more accurately characterized the aggregated nature of coastal fishing effort by recognizing that fishing is not equally distributed across the coastal zone. We generated regional averages based on country-level data. We mapped fishing effort for six regions: West Africa (WA), West Indian Ocean (WIO), Southeast Asia (SEA), Caribbean (CAR), Southwest Atlantic (SWA), and the Eastern Tropical Pacific (ETP) (Figs. 1, 2, 3). These figures illustrate absolute fishing effort density (boat-meters/km^2^) for countries within the regions for all fishing gear. The average fishing effort density in each region was WA, 2.7 boat-meters/km^2^; WIO, 2.2 boat-meters/km^2^; SEA, 3.4 boat-meters/km^2^; ETP, 4.8 boat-meters/km^2^; CAR, 1.2 boat-meters/km^2^; and SWA, 0.3 boat-meters/km^2^.

Looking among regions for each gear category, we identified areas of particularly high fishing density. Most notably, longline densities in the Caribbean region (4.97 boat-meters/km^2^) far exceeded densities in other regions (average  = 0.1 boat-meters/km^2^, ANOVA, F (3, 37)  = 4.05, p = 0.01) (Table 1). Gillnet densities were numerically highest in West Africa (2.78 boat-meters/km^2^) followed by the West Indian Ocean region (1.90 boat-meters/km^2^) (Table 1), although this is based on marginally significant differences (F (5, 21)  = 2.38, p = 0.07). Mixed gear fisheries (defined as fisheries using multiple or unspecified gears) had higher density values globally than other gears, with West Africa and Southeast Asia having the statistically highest densities (8.61 and 7.58 boat-meters/km^2^, respectively, ANOVA, F(5, 117)  = 5.59, p<0.001, Tukey (HSD), p<0.01, Table 1). Trawl densities showed no clear regional patterns or trends (p = 0.61). Finally, trap fisheries were found at higher densities numerically in the Caribbean region, with an average fishing density of 1.89 boat-meters/km^2^ compared to <0.65 boat-meters/km^2^ in other regions, although these differences were not statistically significant (p = 0.26).

Looking across all reported gear types, we also identified countries that had substantially higher fishing densities than other countries in the same region. In West Africa, the equatorial countries of Benin, Togo, and Cameroon had the highest average fishing densities, ranging from 11.1 to 6.5 boat-meters/km^2^ (Table S1). In the West Indian Ocean, the Comoros and Iraq had substantially higher average fishing effort densities, 14.0 and 11.3 boat-meters/km^2^ respectively, than other countries in the region (∼1.2 boat-meters/km^2^ on average) (Table S1). In Southeast Asia, average fishing effort densities were uniformly high across countries (3.4 boat-meters/km^2^), with the highest densities occurring in Bangladesh and the Philippines, with 5.4 and 5.2 boat-meters/km^2^ respectively (Table S1). Honduras, Guatemala and El Salvador were found to have the highest average densities in the Eastern Tropical Pacific region with 36.6, 4.1 and 3.5 boat-meters/km^2^, respectively (Table S1). Fishing density in Honduras was extremely high, although this may be the result of limited spatial data concentrating all fishing effort within the Honduran EEZ inside the Gulf of Fonseca. In the Caribbean region, Bermuda and Haiti had significantly higher average coastal fishing pressure (14.9 and 13.5 boat-meters/km^2^, respectively) than other countries in the region (∼0.7 boat-meters/km^2^) (Table S1). Average densities in the Southwest Atlantic region were relatively low, ranging from 0.6 boat-meters/km^2^ in Brazil to 0.03 boat-meters/km^2^ in Argentina.

### Correlates of fishing pressure

While the goal of our analyses was not to use socioeconomic or physical variables to predict fishing density, we used general regression and generalized linear models to consider whether any of these variables were robust indicators or proxies of national fishing density. Based on the GLM, with model selection performed using Akaike's information criterion (AIC), we found that the best model employed overall population size and length of the coastline as the only significant independent variables, where fishing density increased with population size and decreased with coastline length, i.e., countries with longer coastlines had a greater number of possible cells where fishing could be distributed (df = 2, AIC = 2377.719, p<0.001). However, these variables accounted for very little of the variability in fishing density (df = 5, MS Model = 2.082, adj. r^2^ = 0.10). Fishing effort density demonstrated no significant relationship to Human Development Index (HDI) in these multi-variate models, but considered independently, HDI was significantly and negatively related to fishing density, i.e., fishing density decreased in countries with a High index classification (ANOVA, F(1, 94)  = 3.8726, p = 0.05203).

## Discussion

Fishing pressure is one of the most substantial human impacts on coastal ecosystems. One of the challenges associated with quantifying and then managing sustainable fisheries is the development of a meaningful metric that accurately characterizes fishing pressure across many fishing sectors at national and regional scales. Our derived metric of fishing density, boat-meters/km^2^ is based on empirical data and provides a common measurement for national and regional fisheries assessments. By quantifying fishing density and delimiting fishing grounds across gear types, our analysis highlights coastal areas where fishing pressures are high. These are areas where direct and indirect effects associated with fishing pressure are likely to occur (Figs. 1, 2, 3). Western Equatorial Africa, the Comoros, Iraq, Bermuda, and Haiti had the highest boat densities globally (average density  = 11.7 boat-meters/km^2^), while Bangladesh, the Philippines, Guatemala and El Salvador had moderately high boat densities (average density  = 4.5 boat-meters/km^2^). The Southwest Atlantic had fishing effort densities much lower than other regions. This may reflect the extensive coastline or result from the very limited fishing effort and spatial data that was available from the region, i.e., fisheries with poorly defined spatial limits yielded lower density fisheries.

Regional density differences by gear showed that longlining and fishing with traps (fish and lobster targeted) were most prevalent in the Caribbean region, while gillnets dominated in West Africa and in the West Indian Ocean. In many countries, fishing gear was reported as mixed and target fish were not identified; these mixed gear fisheries had moderately high densities worldwide. These mixed fisheries indicate the prevalence of fishing activities that use multiple types of gear within and among fishing trips. Mixed gear fisheries tended to be present in all regions, particularly in areas with high density small-scale fisheries (i.e., West Africa). This lack of gear definition may obscure differences in regional densities by gear and countries where the effects of fishing activity may be most underestimated are likely to be using mixed gear fisheries. Future efforts to identify specific gears within this category would improve the resolution of fishing density values and distributions by gear type. A number of other factors likely contributed to underestimates of fishing activity. In some regions, there is substantial coastal fishing pressure that is beach-based rather than boat-based, and our method does not include this type of fishing activity [35], [36]. In addition, due to data limitations, the fishing envelopes we present do not include temporal (e.g., seasonal) variability in fishing effort. Rather, the estimates we present provide a ‘snapshot’ of fishing effort across regions.

By mapping fishing effort for different fisheries, we have created a data layer that directly measures the spatially-explicit density of fishing activity across coastal zones. Given the clumped spatial distributions of many target species [37] and associated fishing habitats, fishing effort will be concentrated in areas where catches are highest, leading to clustered and heterogeneous fishing activity across the coastal zone [38], [39]. Restricting our analyses to fishing densities in fished (FMD) rather than all coastal cells (CMD) provides an opportunity to identify areas of particularly high and low fishing effort, where effort deviates from the regional mean. We found that using the spatial extents of fisheries (FMD vs. CMD) identified areas within regions of extreme heterogeneity in effort across the coastal zone (see standard deviation values in Tables 1 & S1). These differences highlight one of the main sources of uncertainty in this analysis and the importance of including the explicit spatial distribution of fishing effort rather than simply tallying vessels or assuming that fishing pressure is equally distributed across coastal waters.

These data provide an important first step to considering cumulative impacts of human activities in marine environments by providing a direct measure of fishing activity, in contrast to previous work that relied on non-spatial proxies of fishing effort [4]. Maps of fishing effort based on recorded descriptions of the spatial extent of a fishery are likely to be a more accurate characterization of fisheries impact within an area. Furthermore, delineating the fishing effort into broad categories also provides information on the differential environmental impacts among gear types, e.g., trawl gear has been documented to degrade benthic habitat [14], and gillnets, as highly non-selective gear, typically lead to high mortality of juvenile target and non-target species [40], [41]. There is empirical support for these gear characterizations. We found gillnet densities to be particularly high in West Africa and in the West Indian Ocean, suggesting that these areas have the potential to exhibit high collateral effects in coastal fisheries. An interview-based rapid fisheries and bycatch assessment in two countries in both of these regions found strong evidence to support the prevalence of high levels of non-target catch [21].

When we examined fishing density as a function of national socioeconomic variables, we found that, as expected, fishing density increased as a function of population size. Length of coastline was also significantly related to fishing densities, i.e., countries with longer coastlines typically had lower fishing densities across that area. Previous research has identified Human Development Index (a national index of life expectancy, literacy and standard of living) as a variable that was significantly related to fishing pressure (25). Although not significant in our multi-variate models, we found that when considered independently from the other variables, countries ranked High on the Human Development Index had lower coastal fishing densities. Conversely, countries with a Low HDI classification were found to have the highest fishing densities in our study. Although the significant relationship we found between HDI and fishing density suggests that coastal fishing pressure may be lower in High HDI countries [5], [25], our results suggest that the development classification may be less important than overall population size and the size of the fishing grounds when characterizing fishing pressure intensity in a region. While the links between population size and coastline length are intuitive and may help provide a coarse characterization of fishing pressure along a coastline, they explained a small proportion of the variability in fishing density and do little to identify which gear types or which coastal areas are likely to experience the highest fishing pressure. For this information, spatially-delimited fishing effort data are needed to accurately map areas of high fishing pressure.

Our approach to mapping fishing density provides a model for how fishing intensity and collateral impacts on coastal zones may be estimated. We found that spatial definition of fishing areas within coastal zones improved our estimates of fishing effort density (see bolded values in FMD vs. CMD, Table S1). Using the FMD method revealed some areas of very high fishing intensity (at least twice as high) that were masked when effort was distributed across all coastal cells (CMD). Our effort metric (boat-meters/km^2^) provided a means to quantify fishing effort density for all fisheries that operate in the coastal zone across fishing sectors (i.e., artisanal vs. industrial). This integration across sectors and gear types is an important first step toward assessing the cumulative direct and indirect effects of fishing in coastal waters.

The integrity and function of coastal ecosystems are challenged by increasing human population pressure on the nearshore environment for food and livelihoods [42]. This trend of population growth intensifies the need for accurate measures of fishing pressure and associated effects in coastal ecosystems. The development of sustainable fisheries management frameworks rely on these data. To date, fishing effort has been largely described using catch metrics and indices of fishing capacity, e.g., horsepower [13]. Catch statistics are far more readily available and do provide information on fishing activities. However, including both estimates of fishing effort and catch statistics will serve as a more accurate basis for evaluating direct and indirect ecological effects of fishing.

## Supporting Information

Table S1 Mean fishing density (boat-meters/km2) for each country within 6 ocean regions. CMD  =  Fishing density distributed among all coastal 1 km grid cells. FMD  =  Fishing density distributed among only those 1 km grid cells that were specified as being fished (data sources - see Appendix S1). SD  =  Standard deviation, n  =  number of fisheries assessed. WA  =  West Africa, WIO  =  West Indian Ocean, SEA  =  Southeast Asia, ETP  =  Eastern Tropical Pacific, CAR  =  Caribbean, and SWA  =  Southwest Atlantic.
(0.17 MB DOC)

Appendix S1 (0.11 MB DOC)

## References

[1] Norse EA (1993). Global marine biological diversity: A strategy for building conservation into decision making..

[2] Hinrichsen D (1996). Coasts in crisis. The earth's most biologically productive habitats are being smothered by development. Only coordinated international action can save them.. Issues Sci Technol.

[3] Kappel CV (2005). Losing pieces of the puzzle: threats to marine, estuarine, and diadromous species.. Front Ecol Environ.

[4] Halpern BS, Walbridge S, Selkoe KA, Kappel CV, Micheli F (2008). A global map of human impact on marine ecosystems.. Science.

[5] Brashares JS, Arcese P, Sam MK, Coppolillo PB, Sinclair ARE (2004). Bushmeat hunting, wildlife declines, and fish supply in West Africa.. Science.

[6] Begossi A (2006). Temporal stability in fishing spots: conservation and co-management in Brazilian artisanal coastal fisheries.. Ecol Soc.

[7] Roberts CM (1997). Ecological advice for the global fisheries crisis.. Trends Ecol Evol.

[8] Caddy JF, Rodhouse PG (1998). Cephalopod and groundfish landings: evidence for ecological change in global fisheries?. Rev Fish Biol Fisher.

[9] Christensen V (1998). Fishery-induced changes in a marine ecosystem: insight from models of the Gulf of Thailand.. J Fish Biol.

[10] Pauly D, Christensen V, Dalsgaard J, Froese R, Torres F (1998). Fishing down marine food webs.. Science.

[11] Sumaila UR, Teh L, Watson R, Tyedmers P, Pauly D (2008). Fuel price increase, subsidies, overcapacity, and resource sustainability.. ICES J Mar Sci.

[12] Madau FA, Idda L, Pulina P (2009). Capacity and economic efficiency in small-scale fisheries: evidence from the Mediterranean Sea.. Mar Policy.

[13] Chuenpagdee R, Liguori L, Palomares MD, Pauly D (2006). Bottom-up, global estimates of small-scale marine fisheries catches.. Fisheries Centre Research Report.

[14] Chuenpagdee R, Morgan LE, Maxwell SM, Norse EA, Pauly D (2003). Shifting gears: assessing collateral impacts of fishing methods in US waters.. Front Ecol Environ.

[15] Lewison RL, Crowder LB, Read AJ, Freeman SA (2004). Understanding impacts of fisheries bycatch on marine megafauna.. Trends Ecol Evol.

[16] Sala E, Aburto-Oropeza O, Reza M, Paredes G, López-Lemus LG (2004). Fishing down coastal food webs in the Gulf of California.. Fisheries.

[17] Huitric M (2005). Lobster and conch fisheries of Belize: a history of sequential exploitation.. Ecol Soc.

[18] Sultana P, Thompson PM (2007). Community based fisheries management and fisher livelihoods: Bangladesh case studies.. Hum Ecol.

[19] Kelleher K (2005). Discards in the world's marine fisheries: an update..

[20] Davies RWD, Cripps SJ, Nickson A, Porter G (2009). Defining and estimating global marine fisheries bycatch.. Mar Policy.

[21] Moore JE, Cox TM, Lewison RL, Read AJ, Bjorkland R (2010). An interview-based approach to assess marine mammal and sea turtle captures in artisanal fisheries.. Biol Conserv.

[22] Mohammed E, Zeller D, Booth S, Mohammed E, Pauly D (2003). Reconstructing fisheries catches and fishing effort for the southeastern Caribbean (1940–2001): general methodology.. From Mexico to Brazil: Central Atlantic Fisheries Catch Trends and Ecosystem Models.

[23] Dunn DC, Stewart K, Bjorkland RH, Haughton M, Singh-Renton S (2010). A regional analysis of coastal and domestic fishing effort in the wider Caribbean.. Fish Res.

[24] Salas SR, Chuenpagdee R, Seijo JC, Charles A (2007). Challenges in the assessment and management of small-scale fisheries in Latin America and the Caribbean.. Fish Res.

[25] Chuenpagdee R, Pauly D (2008). Small is beautiful? A database approach for global assessment of small-scale fisheries: preliminary results and hypotheses.. Am Fish S S.

[26] Lundin CG, Linden O (1993). Coastal ecosystems – attempts to manage a threatened resource.. Ambio.

[27] Stobutzki IC, Silvestre GT, Garces LR (2006). Key issues in coastal fisheries in South and Southeast Asia, outcomes of a regional initiative.. Fish Res.

[28] Bhathal B, Pauly D (2008). ‘Fishing down marine food webs’ and spatial expansion of coastal fisheries in India, 1950–2000.. Fish Res.

[29] Le Pape O, Vigneau J (2001). The influence of vessel size and fishing strategy on the fishing effort for multispecies fisheries in northwestern France.. ICES J Mar Sci.

[30] Bordalo-Machado P (2006). Fishing effort analysis and its potential to evaluate stock size.. Rev Fish Sci.

[31] Piet GJ, Quirijns FJ, Robinson L, Greenstreet SPR (2007). Potential pressure indicators for fishing, and their data requirements.. ICES J Mar Sci.

[32] Snedecor GW, Cochran WG (1989). Statistical methods, eighth edition..

[33] United Nations Development Programme (2007). Human Development Report 2007/2008.. P Macmillan.

[34] International Monetary Fund (2009). World Economic Outlook Database..

[35] MacKenzie CL, Buesa RJ (2006). Vida de los pescadores costeros del Pacífico desde México a Perú y su dependencia de la recolecta de conchas (*Anadara* spp.), almejas (*Polymesoda* spp.), ostiones (*Crassostrea* spp., *Ostreola* spp.), camarones (*Penaeus* spp.), cangrejos (*Callinectes* spp.), y la pesca de peces de escama en Los Manglares. (The fishermen's lives in Pacific coast villages from Mexico to Peru, supported by landings of mangrove cockles (*Anadara* spp.), clams (*Polymesoda* spp.), oysters (*Crassostrea* spp., *Ostreola* spp.), shrimp (*Penaeus* spp.), crabs (*Callinectes* spp.), and finfish.. US Dept Commerce, Northeast Fish Sci Cent Ref Doc.

[36] Purcell SW, Lovatelli A, Vasconcellos M, Yimin Y (2010). Managing sea cucumber fisheries with an ecosystem approach.. FAO Fisheries and Aquaculture Technical Paper. No. 520. Rome, FAO.

[37] Greenstreet SPR, Spence FE, McMillan JA (1999). Fishing effects in northeast Atlantic shelf seas: patterns in fishing effort, diversity and community structure. V. Changes in structure of the North Sea groundfish species assemblage between 1925 and 1996.. Fish Res.

[38] Booth AJ (2000). Incorporating the spatial component of fisheries data into stock assessment models.. ICES J Mar Sci.

[39] Verdoit M, Pelletier D, Bellail B (2003). Are commercial logbook and scientific CPUE data useful for characterizing the spatial and seasonal distribution of exploited populations? The case of the Celtic Sea whiting.. Aquat Liv Resour.

[40] He P (2006). Gillnets, gear design, fishing performance and conservation challenges.. Mar Technol Soc J.

[41] Gilman E, Gearhart J, Price B, Eckert S, Milliken H (2010). Mitigating sea turtle by-catch in coastal passive net fisheries.. Fish Fish.

[42] Unsworth RKF, Cullen LC (2010). Recognising the necessity for Indo-Pacific seagrass conservation.. Cons Lett.

