# Supplementary material for: Characterizing Fishing Effort and Spatial Extent of Coastal Fisheries
Source: PLoS One. 2010 Dec 29;5(12):e14451. doi: 10.1371/journal.pone.0014451 (PMC3012055; doi:10.1371/journal.pone.0014451)
Supplement: Table S1 — Mean fishing density (boat-meters/km2) for each country within 6 ocean regions. CMD = Fishing density distributed among all coastal 1 km grid cells. FMD = Fishing density distributed among only those 1 km grid cells that were specified as being fished (data sources - see Appendix S1). SD = Standard deviation, n = number of fisheries assessed. WA = West Africa, WIO = West Indian Ocean, SEA = Southeast Asia, ETP = Eastern Tropical Pacific, CAR = Caribbean, and SWA = Southwest Atlantic. (0.17 MB DOC) [file pone.0014451.s001.doc]

Table S1. Mean fishing density (boat-meters/ km^2^) for each country within 6 ocean regions. CMD = Fishing density distributed among all coastal 1 km grid cells. FMD = Fishing density distributed among only those 1 km grid cells that were specified as being fished (data sources – see Appendix 1). SD = Standard deviation, n = number of fisheries assessed. WA = West Africa, WIO = West Indian Ocean, SEA = Southeast Asia, ETP = Eastern Tropical Pacific, CAR = Caribbean, and SWA = Southwest Atlantic.

| **Region** | **Country** | **n** | **CMD** | **SD** | **FMD** | **SD** | **Region** | **Country** | **n** | **CMD** | **SD** | **FMD** | **SD** |
| --- | --- | --- | --- | --- | --- | --- | --- | --- | --- | --- | --- | --- | --- |
| WA | South Africa | 4 | 0.02 | 0.03 | 0.03 | 0.04 | SEA | Vietnam | 3 | 1.78 | 4.96 | 1.86 | 5.05 |
| WA | Namibia | 6 | 0.03 | 0.01 | 0.03 | 0.01 | SEA | Thailand | 1 | 1.79 | 1.95 | 3.18 | 1.52 |
| WA | Guinea Bissau | 2 | 0.05 | 0.08 | 0.05 | 0.08 | SEA | Sri Lanka | 3 | 3.55 | 3.98 | 4.00 | 4.00 |
| WA | Nigeria | 3 | 0.21 | 0.71 | 0.28 | 0.80 | SEA | India | 2 | 3.50 | 2.93 | 4.48 | 2.56 |
| WA | Gabon | 3 | 0.37 | 0.87 | 0.38 | 0.87 | SEA | Taiwan | 1 | 1.65 | 2.68 | **4.75** | 2.46 |
| WA | Angola | 4 | 0.43 | 1.34 | 0.44 | 1.35 | ETP | Peru | 2 | 0.47 | 0.23 | 0.48 | 0.23 |
| WA | Sao Tome and Principe | 4 | 0.79 | 3.80 | 0.79 | 3.81 | ETP | Nicaragua | 3 | 0.37 | 1.19 | **0.75** | 1.61 |
| WA | Cape Verde | 2 | 0.10 | 0.32 | **0.83** | 0.47 | ETP | Costa Rica | 5 | 0.78 | 1.87 | 0.79 | 1.88 |
| WA | Côte d'Ivoire | 3 | 0.95 | 3.86 | 0.99 | 3.94 | ETP | Chile | 15 | 0.27 | 2.34 | **0.81** | 4.05 |
| WA | Republic of Congo | 2 | 1.16 | 2.14 | 1.18 | 2.15 | ETP | Panama | 3 | 0.61 | 0.55 | 1.05 | 0.24 |
| WA | Mauritania | 4 | 1.14 | 3.16 | 1.19 | 3.22 | ETP | Mexico | 15 | 1.07 | 1.22 | 1.08 | 1.22 |
| WA | Sierra Leone | 2 | 1.32 | 3.04 | 1.97 | 3.54 | ETP | Ecuador | 10 | 1.27 | 2.67 | 1.29 | 2.68 |
| WA | Liberia | 2 | 1.24 | 3.19 | 2.06 | 3.91 | ETP | Colombia | 3 | 0.70 | 1.28 | **2.09** | 1.40 |
| WA | Morocco | 8 | 2.51 | 3.02 | 2.77 | 3.05 | ETP | El Salvador | 3 | 3.32 | 2.66 | 3.47 | 2.63 |
| WA | Senegal | 3 | 3.28 | 7.68 | 3.32 | 7.71 | ETP | Guatemala | 3 | 1.65 | 2.69 | **4.12** | 2.82 |
| WA | Equatorial Guinea | 1 | 0.28 | 1.03 | **3.35** | 1.63 | ETP | Honduras | 1 | 30.95 | 17.14 | 36.62 | 11.84 |
| WA | Ghana | 6 | 3.60 | 8.97 | 3.75 | 9.12 | CAR | Belize | 2 | 0.16 | 0.26 | **0.53** | 0.15 |
| WA | Gambia | 3 | 3.09 | 6.91 | 3.94 | 7.59 | CAR | Barbados | 3 | 0.68 | 0.56 | 0.68 | 0.56 |
| WA | Guinea | 1 | 1.09 | 3.63 | **4.95** | 6.40 | CAR | Guadeloupe and Martinique | 2 | 0.49 | 1.40 | 0.80 | 1.72 |
| WA | Cameroon | 2 | 5.78 | 12.16 | 6.53 | 12.73 | CAR | Costa Rica | 1 | 0.18 | 0.40 | **0.94** | 0.32 |
| WA | Togo | 1 | 1.79 | 4.66 | **10.36** | 6.05 | CAR | Jamaica | 2 | 0.42 | 0.92 | **1.84** | 1.02 |
| WA | Benin | 2 | 1.74 | 4.82 | **11.10** | 6.66 | CAR | Antigua and Barbuda | 2 | 0.15 | 0.74 | **2.65** | 1.63 |
| WIO | Seychelles | 3 | 0.02 | 0.03 | 0.02 | 0.03 | CAR | Dominica | 2 | 1.00 | 10.19 | **2.85** | 17.08 |
| WIO | Madagascar | 2 | 0.01 | 0.02 | 0.05 | 0.02 | CAR | Puerto Rico and US Virgin Islands | 7 | 0.50 | 6.57 | **5.86** | 21.66 |
| WIO | Egypt | 3 | 0.09 | 0.07 | 0.09 | 0.06 | CAR | Haiti | 1 | 0.61 | 2.88 | **13.48** | 3.27 |
| WIO | Somalia | 4 | 0.17 | 0.17 | 0.17 | 0.17 | CAR | Bermuda | 1 | 0.01 | 0.64 | **14.89** | 14.13 |
| WIO | Qatar | 1 | 0.20 | 0.10 | 0.21 | 0.09 | CAR | Colombia | 3 | 0.02 | 0.03 | 0.02 | 0.03 |
| WIO | Maldives | 1 | 0.28 | 0.14 | 0.29 | 0.14 | CAR | Bahamas | 1 | 0.01 | 0.02 | **0.03** | 0.01 |
| WIO | Djibouti | 1 | 0.11 | 0.16 | **0.32** | 0.10 | CAR | French Guiana | 3 | 0.04 | 0.02 | 0.04 | 0.02 |
| WIO | Eritrea | 2 | 0.14 | 0.21 | **0.43** | 0.08 | CAR | Netherlands Antilles | 2 | 0.05 | 0.01 | 0.05 | 0.01 |
| WIO | Mauritius | 3 | 0.17 | 0.46 | **0.55** | 0.69 | CAR | Turks and Caicos Islands | 2 | 0.02 | 0.04 | **0.10** | 0.02 |
| WIO | Saudi Arabia | 4 | 0.55 | 0.59 | 0.56 | 0.59 | CAR | Cuba | 3 | 0.12 | 0.19 | 0.12 | 0.19 |
| WIO | Iran | 3 | 0.49 | 0.22 | 0.59 | 0.02 | CAR | St. Maarten | 3 | 0.04 | 0.21 | **0.15** | 0.40 |
| WIO | Yemen | 4 | 0.70 | 0.37 | 0.70 | 0.37 | CAR | Honduras | 5 | 0.11 | 0.13 | 0.18 | 0.13 |
| WIO | Mozambique | 4 | 0.60 | 0.80 | 0.79 | 0.83 | CAR | Panama | 2 | 0.10 | 0.12 | 0.19 | 0.10 |
| WIO | Kenya | 3 | 0.78 | 1.42 | 1.06 | 1.57 | CAR | Suriname | 6 | 0.27 | 0.51 | 0.27 | 0.51 |
| WIO | Kuwait | 7 | 1.20 | 0.98 | 1.21 | 0.97 | CAR | St. Vincent | 3 | 0.30 | 0.23 | 0.30 | 0.23 |
| WIO | Oman | 1 | 1.27 | 0.75 | 1.33 | 0.70 | CAR | Montserrat | 3 | 0.06 | 0.40 | **0.30** | 0.87 |
| WIO | United Arab Emirates | 4 | 1.38 | 0.61 | 1.38 | 0.61 | CAR | Trinidad and Tobago | 8 | 0.30 | 1.11 | 0.31 | 1.12 |
| WIO | Sudan | 2 | 0.06 | 0.46 | **2.84** | 1.63 | CAR | Dominican Republic | 1 | 0.32 | 0.12 | 0.33 | 0.09 |
| WIO | Tanzania | 2 | 0.87 | 1.45 | **3.12** | 0.76 | CAR | Grenada | 7 | 0.34 | 0.82 | 0.35 | 0.83 |
| WIO | Bahrain | 4 | 2.97 | 2.03 | 3.64 | 1.61 | CAR | Venezuela | 6 | 0.35 | 0.35 | 0.35 | 0.35 |
| WIO | Pakistan | 4 | 1.92 | 4.77 | **5.34** | 6.70 | CAR | Guyana | 7 | 0.27 | 0.93 | 0.37 | 1.07 |
| WIO | Iraq | 2 | 6.01 | 6.61 | 11.27 | 4.76 | CAR | Nicaragua | 5 | 0.39 | 0.17 | 0.43 | 0.12 |
| WIO | The Comoros | 1 | 0.66 | 3.06 | **13.97** | 3.38 | CAR | Saint Kitts and Nevis | 4 | 0.43 | 1.34 | 0.43 | 1.34 |
| SEA | Indonesia | 4 | 1.58 | 2.76 | **5.07** | 2.61 | CAR | St. Lucia | 4 | 0.44 | 0.74 | 0.44 | 0.74 |
| SEA | Philippines | 15 | 5.19 | 9.26 | 5.20 | 9.26 | CAR | Anguilla | 5 | 0.08 | 0.37 | **0.50** | 0.78 |
| SEA | Bangladesh | 3 | 5.35 | 7.40 | 5.38 | 7.41 | SWA | Argentina | 5 | 0.03 | 0.08 | 0.03 | 0.08 |
| SEA | Cambodia | 2 | 0.86 | 1.24 | 0.89 | 1.25 | SWA | Brazil | 3 | 0.50 | 0.20 | 0.57 | 0.08 |
| SEA | Malaysia | 13 | 1.02 | 1.44 | 1.03 | 1.44 | SWA | Uruguay | 3 | 0.13 | 0.49 | 0.14 | 0.51 |
| SEA | Myanmar | 4 | 1.00 | 0.51 | 1.06 | 0.46 |  |  |  |  |  |  |  |
