# Supplementary material for: Characterizing Fishing Effort and Spatial Extent of Coastal Fisheries
Source: PLoS One. 2010 Dec 29;5(12):e14451. doi: 10.1371/journal.pone.0014451 (PMC3012055; doi:10.1371/journal.pone.0014451)
Supplement: Appendix S1 — (0.11 MB DOC) [file pone.0014451.s002.doc]

**Supporting Information - APPENDIX 1**

**Additional effort metric development details**

To develop the fishing density metric (boat-meters/ km^2^) for as many fisheries as possible, it was necessary to make some assumptions or rely on supplementary data. For example, for fisheries with no boat lengths reported, we estimated boat length following a 2-step process. In the first step, we identified or classified the development level of a fishery. For fisheries with no specified development level, we evaluated other available information (e.g., number of boats, size of boats and fishing grounds information) and used these related measures to assign development level. We then sorted the data by region, development level and gear type to assign a boat size. For example, if one industrial gillnet fishery was missing boat length information, we looked at all the industrial gillnet fisheries within the region, calculated the average and standard deviation for the size of those boats, and then used that average to fill in the missing boat length information. If gear-specific information was not available, then we used the region-specific, development level-specific boat length average.

For fisheries with no defined spatial extents, we used related information, e.g., known distribution of target species, known distribution of associated habitat, or type of fishing gear used to identify the fishing grounds. For example, all shrimp trawls and trap fisheries were limited to shelf waters (-200 m depth). If artisanal fisheries included fish as well as shrimp as the target species, they were limited to 50 km from shore. Pelagic fisheries without known spatial extent data within the coastal zone were excluded because we were unable to characterize the percent of the fishing effort inside vs. outside the coastal zone. For other fisheries known to occur within the coastal zone and extend out beyond the coastal limits defined here (either -200 m depth or 50 km distance), effort was distributed equally throughout the relevant cells, however, only the coastal component of these fisheries was mapped and analyzed.

**Fishing Effort Data Sources**

Acheampong A (1997) Coherence between EU fisheries agreements and EU development cooperation: The case of West Africa (EDCPM Working Paper No. 52). Maastricht ECDPM. 28 pp.

Adpesca, AECI (2002) Diagnostico de la actividad pesquera y acuícola. Managua, Nicaragua. 390 pp.

Aguilar FW, Revelo D, Coello J, Cajas W, Ruiz M, et al. (2006) Desembarques artesanales de tiburones y rayas en los principales puertos pesqueros del ecuador durante 2006. 19 pp.

Ambrose EE, Solarin BB, Isebor CE, Williams AB (2004) Assessment of fish by-catch species from coastal artisanal shrimp beam trawl fisheries in Nigeria. Fish Res 71:125-132.

Anonymous (1999) Barbados Fisheries Management Plan, The Fisheries Division, Ministry of Agriculture and Rural Development.

Anonymous (2005) Annual Fisheries Statistics. Department of Fisheries Sabah, Kota Kinabalu, Sabah.

Arata J, Hucke-Gaete R (2005) Pesca incidental de aves y mamíferos: Devastación marina. Oceana. Oficina para América del Sur y Antártica. 81 pp.

Arauz R (1999) Description of the eastern Pacific high-seas longline and coastal gillnet swordfish fisheries of South America, including sea turtle interactions, and management recommendations. Sea Turtle Restoration Project Turtle Island Restoration Network. Drexel University. 33 pp.

Arauz R, Rodriguez O, Vargas R, Segura A (2000) Incidental capture of sea turtles by Costa Rica's longline fleet, pp. 62-64. In Proceedings from the 19th Annual Symposium on Sea Turtle Biology and Conservation, South Padre Island, TX, USA.

Arauz RM, Vargas R, Naranjo I, Gamboa C (1998) Analysis of the incidental capture and mortality of sea turtles in the shrimp fleet of Pacific Costa Rica. U.S. Dep. Commer. NOAA Tech. Memo. NMFS-SEFSC-415. 5 pp.

Arriaga L, Martinez J (2002) Plan de ordenamiento de la pesca y acuicultura del Ecuador. Subsecretaria de recursos pesqueros. 116 pp.

Aubray R (1975) The fisheries of Ethiopia: An economic study. Rome, FAO (mimeo). Cited in Project GloBAL. 2007. Project GloBAL Country Profile: Eritrea. Duke Center for Marine Conservation, Duke University Marine Laboratory, Beaufort, NC.

Baloaños M (2005) Characterisation of the Costa Rican semi-industrial coastal shrimp trawling fishery and the small scale artisanal shrimp trawling fishery. Document presented as part of the Reduction of Environmental Impact from Tropical Shrimp Trawling, Through the Introduction of By-Catch Reduction Technologies and the Change of Management Project. Project GEF-UNEP-FAO-INCOPESCA. Project No. EP/GLO/201/GEF. FAO, Costa Rica Office. 41 pp.

Bannerman P, Quartey R (2005) Report on the observations of commercial light fishing operation in Ghana, February - June 2004. The Marine Fisheries Research Division (MFRD), Tema. 8 pp.

Beltrán-Turriago CS (2001) Promoción de la ordenación de la pesca costera. 2. Aspectos socioeconómicos y técnicos de la pesca artesanal en El salvador, Costa Rica, Panamá, Ecuador y Colombia. FAO Circular de Pesca. No. 957/2. Roma, FAO. 71p.

Beltrán-Turriago CS, Villaneda Jiménez AA (2000) Perfil de la pesca y la acuicultura en Colombia. Instituto Nacional de Pesca y Acuicultura - INPA, Subdirección de Investigaciones. Santafé de Bogotá. 29 pp.

Carvajal Rodríguez JM (2006) Generalidades del sector pesquero de Costa Rica. Instituto Costarricense de Pesca y Acuicultura. Puntarenas, Costa Rica. 10 pp.

Charuau A, Medley PA (2001) French Guiana, Snapper Fishery. FAO Fisheries Report 651, pp 77-80. Food and Agriculture Organization of the United Nations. Rome.

Charuau A, Cochrane K, Die D, Larez A, Marcano LA, et al. (2001) Regional assessment of red snapper, *Lutjanus* *purpureus*. FAO Fisheries Report 651, pp. 15-21. Food and Agriculture Organization of the United Nations. Rome.

Chavance P, Tous P, Ould Sidi Ould Beibou E, Wagne OH (2006) Une estimation de la production récente de la pêche artisanale Mauritanienne. Bulletin Scientifique de l'IMROP, Nouadhibou, 30: 25-36 cited in Gascuel, D., P. Labrosse, B. Meissa, M.O. Taleb Sidi and S. Guénette. 2007. Decline of demersal resources in North-West Africa: an analysis of Mauritanian trawl-survey data over the past 25 years. Afr J Mar Sci 29(3): 331-345.

Chicaiza D (2006) Principales aspectos biológicos pesqueros del camarón pomade (*Protrachypene* *preciupa*) en la zona de Posorja durante 2005. Instituto Nacioal de Pesca: Informe Téchnico IRBA 1-83-04: 4pp.

Chuenpagdee R, Liguori L, Palomares MD, Pauly D (2006) Bottom-up, global estimates of small-scale marine fisheries catches. Fisheries Centre Research Report 14(8): 1-112.

CI DECR (2004) Turks and Caicos Islands Department of Environment and Coastal Resources Website. http://www.environment.tc/intro.htm. Last Accessed September 11, 2006.

CONAPESCA (2004) Anuario estadístico de acuacultura y pesca. Secretaría de Agricultura, Ganadería, Desarrollo Rural, Pesca y Alimentación, Mexico.

Correa J, Chicaiza D, Mendívez W, González J, García-Sáenz R (2007) La pesquería de arrastre de camarón marino en Ecuador durante el 2006. Instituto Nacional de Pesca. PowerPoint presentation.

CPPS (2001) Estudio sobre el impacto socio-económico de la pesca artisanal en los estados miembros de la comisión permanente del Pacífico Sur. 33 pp.

CPPS (2005) II Taller Tecnico-Cientifico sobre el pez espada en el Pacifico Sudeste. Valparaíso, 16-17 mayo de 2005. Informe de la reunion. 119 pp.

CRFM (2006) http://www.caricom-fisheries.com/members/dominica.asp.

D'Agrossa C, Lennert-Cody CE, Vidal O (2000) Vaquita bycatch in Mexico's artisanal gillnet fisheries: Driving a small population to extinction. Conserv Biol 14(4): 1110-1119.

de Sousa LP, Brito A, Abdula S, Caputi N (2006) Research assessment for the management of the industrial shallow-water multi-species shrimp fishery in Sofala Bank in Mozambique. Fish Res 77: 207-219.

Delamare A (2005) Estimation des captures accidentelles de tortues marines par les fileyeurs de la pêche côtière en Guyane. MSc dissertation, Agrocampus de Rennes.

Delcroix E (2003) Etude des captures accidentelles de tortues marines par la pêche maritime dans les eaux de l'archipel guadeloupéen. Maîtrise des Sciences et Techniques Aménagement et Environnement à Metz Maître de stage Johan CHEVALIER Tuteur universitaire J.C Moreteau. 84 pp.

Diaz N, Doray M, Reynal L, Gervain P, Carpentier A (2002) Pêche des poissons pélagiques hauturiers et développement des DCP ancrés en Guadeloupe. FAO Fisheries Report 683: 39-54.

Dilrosun F (2002) Progress report on Curacao fishery monitoring programme (November 2000 - July 2001). pp. 9-20 in FAO Fisheries Report No. 683, Supplement. Western Central Atlantic Fishery Commission: National reports and technical papers presented at the first meeting of the WECAFC Ad Hoc Working Group on the development of sustainable moored fish aggregating device fishing in the Lesser Antilles. Le Robert, Martinique, 8-11 October 2001. Food and Agriculture Organization of the United Nations. Rome.

Dilrosun F (2000) Monitoring the Saba Bank fishery. BOM Project N5. Department of Public Health and Environmental Hygiene Environmental Division. Curaçao, Netherlands Antilles. 56 pp.

Domingo A, Bugoni L, Prosdocimi L, Miller P, Laporta M, et al. (2006) The impact generated on sea turtles by fisheries in the Southwest Atlantic Ocean. WWF Programa Marino para Latinoamerica y el Caribe, San Jose, Costa Rica.

Domingo A, Sales G, Giffoni B, Millar P, Laporta M, et al. (2006). Captura incidental de tortugas con palangre pelágico en el atlántico por las flotas de Brasil y Uruguay. Col Vol Sci Pap ICCAT 59: 992-1002.

European Commission (EC) (2005) About the common fisheries policy: Comoros Islands. Unit for Information and communication. European Commission. Available at: http://ec.europa.eu/fisheries/cfp/external_relations/bilateral_agreements/comoros_en.htm. Accessed on: October 10, 2006.

FAO (2003) FAO Country Profile: Cuba. Food and Agriculture Organization of the United Nations, Rome.

FAO (2005) FAO Country Profile: Barbados. Food and Agriculture Organization of the United Nations, Rome.

FAO (2005) FAO Country Profile: Belize. Food and Agriculture Organization of the United Nations, Rome.

FAO (2005) FAO Country Profile: Brazil. Food and Agriculture Organization of the United Nations, Rome.

FAO (2005) FAO Country Profile: Cambodia. Food and Agriculture Organization of the United Nations, Rome.

FAO (2005) FAO Country Profile: Colombia. Food and Agriculture Organization of the United Nations, Rome.

FAO (2005) FAO Country Profile: Costa Rica. Food and Agriculture Organization of the United Nations, Rome.

FAO (2005) FAO Country Profile: Dominica. Food and Agriculture Organization of the United Nations, Rome.

FAO (2005) FAO Country Profile: Haiti. Food and Agriculture Organization of the United Nations, Rome.

FAO (2005) FAO Country Profile: Honduras. Food and Agriculture Organization of the United Nations, Rome.

FAO (2005) FAO Country Profile: Nicaragua. Food and Agriculture Organization of the United Nations, Rome.

FAO (2005) FAO Country Profile: Nicaragua. Food and Agriculture Organization of the United Nations, Rome.

FAO (2005) FAO Country Profile: Panama. Food and Agriculture Organization of the United Nations, Rome.

FAO (2005) FAO Country Profile: St. Kitts and Nevis. Food and Agriculture Organization of the United Nations, Rome.

FAO (2005) FAO Country Profile: St. Lucia. Food and Agriculture Organization of the United Nations, Rome.

FAO (2005) FAO Country Profile: St. Vincent. Food and Agriculture Organization of the United Nations, Rome.

FAO (2005) FAO Country Profile: Trinidad and Tobago. Food and Agriculture Organization of the United Nations, Rome.

FAO (2005) FAO Country Profile: Venezuela. Food and Agriculture Organization of the United Nations, Rome.

FAO (2005) FAO Country Profile: Viet Nam. Food and Agriculture Organization of the United Nations, Rome.

FAO (2005) Information on Fisheries Management in Jamaica. Food and Agriculture Organization of the United Nations, Rome.

FAO (2000) FAO Country Profile: Namibia. Food and Agriculture Organization of the United Nations, Rome.

FAO (2005) FAO Country Profile: Nigeria. Food and Agriculture Organization of the United Nations, Rome.

FAO (1997) FAO Country Profile: Mauritania. Food and Agriculture Organization of the United Nations, Rome.

FAO (1998) FAO Country Profile: Bahrain. Food and Agriculture Organization of the United Nations, Rome.

FAO (1998) FAO Country Profile: Madagascar. Food and Agriculture Organization of the United Nations, Rome.

FAO (1998) FAO Country Profile: Yemen. Food and Agriculture Organization of the United Nations, Rome.

FAO (1998) Report of the Workshop on the Review of the Pelagic Surveys off Northwest Africa in the 1990s. Bergen, Norway, 28 September - 2 October 1998.

FAO (2000) FAO Country Profile: Ghana. Food and Agriculture Organization of the United Nations, Rome.

FAO (2000) FAO Country Profile: Morocco. Food and Agriculture Organization of the United Nations, Rome.

FAO (2001) FAO Country Profile: Guinea Bissau. Food and Agriculture Organization of the United Nations, Rome.

FAO (2001) FAO Country Profile: Pakistan. Food and Agriculture Organization of the United Nations, Rome.

FAO (2001) Tropical shrimp fisheries and their impact on living resources. Shrimp fisheries in Asia: Bangladesh, Indonesia and the Philippines; in the Near East: Bahrain and Iran; in Africa: Cameroon, Nigeria and the United Republic of Tanzania; in Latin America: Colombia, Costa Rica, Cuba, Trinidad and Tobago, and Venezuela. FAO Fisheries Circular. No. 974. Rome, FAO. 378pp.

FAO (2002) FAO Country Profile: United Arab Emirates. Food and Agriculture Organization of the United Nations, Rome.

FAO (2002) FAO Country Profile: Eritrea. Food and Agriculture Organization of the United Nations, Rome.

FAO (2002) Western Central Atlantic Fishery Commission: Report of the first meeting of the WECAFC Ad Hoc Working Group on the development of sustainable moored fish aggregating device fishing in the Lesser Antilles. Le Robert, Martinique, 8-11 October 2001. FAO Fisheries Report No.683. Food and Agriculture Organization of the United Nations. Rome. 28 pp.

FAO (2003) FAO Country Profile: Comoros. Food and Agriculture Organization of the United Nations, Rome.

FAO (2003) FAO Country Profile: Iran. Food and Agriculture Organization of the United Nations, Rome.

FAO (2003) FAO Country Profile: Kuwait. Food and Agriculture Organization of the United Nations, Rome.

FAO (2003) FAO Country Profile: Saudi Arabia. Food and Agriculture Organization of the United Nations, Rome.

FAO (2003) FAO Country Profile: Seychelles. Food and Agriculture Organization of the United Nations, Rome.

FAO (2003) FAO Country Profile: United Arab Emirates. Food and Agriculture Organization of the United Nations, Rome.

FAO (2003) Report of the FAO Working Group on the Assessment of Small Pelagic Fish off Northwest Africa. Agadir, Morocco, 31 March-10 April.

FAO (2003) FAO Country Profile: Somalia. Food and Agriculture Organization of the United Nations, Rome.

FAO (2004) FAO Country Profile: Oman. Food and Agriculture Organization of the United Nations, Rome.

FAO (2005) FAO Country Profile: Djibouti. Food and Agriculture Organization of the United Nations, Rome.

FAO (2005) FAO Country Profile: Mauritius. Food and Agriculture Organization of the United Nations, Rome.

FAO (2005) FAO Country Profile: Qatar. Food and Agriculture Organization of the United Nations, Rome.

FAO (2005) FAO Country Profile: Sao Tome and Principe. Food and Agriculture Organization of the United Nations, Rome.

FAO (2005) FAO Country Profile: South Africa. Food and Agriculture Organization of the United Nations, Rome.

FAO (2005) FAO Country Profile: South Africa. Food and Agriculture Organization of the United Nations, Rome.

FAO (2005) FAO Country Profile: Tanzania. Food and Agriculture Organization of the United Nations, Rome.

FAO (2005) FAO Country Profile: Togo. Food and Agriculture Organization of the United Nations, Rome.

FAO (2005) FAO Country Profile: Cameroon. Food and Agriculture Organization of the United Nations, Rome.

FAO (2005) FAO Country Profile: Oman. Food and Agriculture Organization of the United Nations, Rome.

FAO (2006) FAO Country Profile: Kenya. Food and Agriculture Organization of the United Nations, Rome.

FAO (2006) South West Indian Ocean Fisheries Commission/Commission des pêches pour le sud-ouest de l'océan Indien. Report of the first session of the Scientific Committee. Dar es Salaam, United Republic of Tanzania, 31 May-3 June 2006. FAO Fisheries Report/FAO Rapport sur les pêches. No. 806. Rome, FAO. 2006. 79pp.

FAO (2007) FAO Country Profile: Angola. Food and Agriculture Organization of the United Nations, Rome.

FAO (2007) FAO Country Profile: Cape Verde. Food and Agriculture Organization of the United Nations, Rome.

FAO (2007) FAO Country Profile: Gabon. Food and Agriculture Organization of the United Nations, Rome.

FAO (2007) FAO Country Profile: Gambia. Food and Agriculture Organization of the United Nations, Rome.

FAO (2007) FAO Country Profile: Mozambique. Food and Agriculture Organization of the United Nations, Rome.

FAO (2007) FAO Country Profile: Panama. Food and Agriculture Organization of the United Nations, Rome.

FAO (2008) Costa Rica panorama general del sector pesquero (de la NFSO). http://www.fao.org/fishery/countrysector/FI-CP_CR/es. Viewed June 2008.

FAO (2008) El Salvador panorama general del sector pesquero (de la NFSO). http://www.fao.org/fishery/countrysector/FI-CP_SV/es. Viewed on June 20th 2008.

FAO (2008) FAO Country Profile: Benin. Food and Agriculture Organization of the United Nations, Rome.

FAO (2008) FAO Country Profile: Cote d'Ivoire. Food and Agriculture Organization of the United Nations, Rome.

FAO (2008) FAO Country Profile: Guinea. Food and Agriculture Organization of the United Nations, Rome.

FAO (2008) FAO Country Profile: Senegal. Food and Agriculture Organization of the United Nations, Rome.

FAO (2008) FAO Country Profile: Sierra Leone. Food and Agriculture Organization of the United Nations, Rome.

FAO (2008) Honduras - Visión general del sector pesquero nacional (de la NFSO). http://www.fao.org/fishery/countrysector/FI-CP_HN/es. Viewed June 2008.

FAO (2008) Nicaragua panorama general del sector pesquero (de la NFSO). http://www.fao.org/fishery/countrysector/FI-CP_NI/es. Viewed June 2008.

FAO (2009) FAO Country Profile: Chile. Food and Agriculture Organization of the United Nations, Rome.

FAO (2003-2009) Fisheries Topics: Statistics. Fisheries statistics and information. In: FAO Fisheries and Aquaculture Department [online]. Rome. Updated 2006 15 09.

FAO-OSPESCA (2006) Mejoramiento de los sistemas de información y recolección de datos pesqueros para América Central y el Caribe: Informe del Taller FAO/OSPESCA sobre el mejoramiento de los sistemas de información y recolección de datos pesqueros, San Salvador, El Salvador, 23-26 de enero 2006. Volumen 1: Informe del Taller, 41 pp.

Faraj A, Bez N (2007) Spatial considerations for the Dakhla stock of Octopus vulgaris: indicators, patterns, and fisheries interactions. ICES J Mar Sci 64: 1820-1828.

FDF (1995) Fisheries Statistics of Nigeria, third ed. Published by the Federal Department of Fisheries, Abuja, 51 pp. Cited in Ambrose EE, Solarin BB, Isebor CE, Williams AB (2004) Assessment of fish by-catch species from coastal artisanal shrimp beam trawl fisheries in Nigeria. Fish Res 71: 125-132.

FIGIS data. W. Africa fishing vessel class and numbers 1970-1995.

Fisheries Division (1999) National Report of Trinidad and Tobago: The shrimp and groundfish fisheries of Trinidad and Tobago. In: National reports presented and stock assessment reports prepared at the CFRAMP/FAO/DANIDA Stock assessment workshop on the shrimp and groundfish fisheries on the Guiana-Brazil shelf. FAO/Western Central Atlantic Fishery Commission. Port-of-Spain, Trinidad and Tobago, 7-18 April 1997. FAO Fisheries Report No. 600. Rome, FAO. 1999. 200 pp.

Fisheries Global Information System (FIGIS) (2002) FIGIS - FIRMS Methodological Workshop Report. 1-5 July 2002, FAO Headquarters, Rome. 46 pp.

Fisheries Policy and Economics Division, BFAR (2005) Philippine Fisheries Profile 2005. Republic of the Philippines, Bureau of Fisheries and Agriculture. Quezon City. 32 pp.

Franquesa R, Malouli IM, Alarcón JA (2001) Feasibility assessment for a database on socio-economic indicators for Mediterranean fisheries. Studies and Reviews. General Fisheries Commission for the Mediterranean. No. 71. Rome, FAO. 55p.

Gandini P, Frere E (2006) Spatial and temporal patterns in the bycatch of seabirds in the Argentinian longline fishery. Fish Bull 104: 482-485.

Garrison VH, Rogers CS, Beets J, Friedlander AM (2004) The habitats exploited and the species trapped in a Caribbean island trap fishery. Environ Biol Fish 71: 247-260.

Gascuel D, Labrosse P, Meissa B, Taleb Sidi MO, Guénette S (2007) Decline of demersal resources in North-West Africa: an analysis of Mauritanian trawl-survey data over the past 25 years. Afr J Mar Sci 29(3): 331-345.

Gbaguidi A (1993) Small scale fishery for sardinella in Benin. Chargée de Statistiques Service des Pêches du Bénin. 9 pp.

Gobert B, Berthou P, Lopez E, Lespagnold P, Oqueli Turciose MD, et al. (2005) Early stages of snapper-grouper exploitation in the Caribbean (Bay Islands, Honduras). Fish Res 73(1-2): 159-169.

Gobierno de Chile, Subsecretaría de Pesca (2006) Informe sectorial de pesca y acuicultura. 22 pp.

Government of Pakistan (2002) Fisheries statistics, 1996-2001 (Annexure I). 2 pp.

Grant S (2006) Managing small-scale fisheries in the Caribbean: the surface longline fishery in Gouyave, Grenada. Doctoral dissertation. University of Manitoba, Natural Resources Institute University. Winnipeg, Manitoba. 275 pp.

Griffith L (1998) Under threat from the super-net. WWF International-Newsroom-Features. 3 pp.

Guía Indicativa (2005) Nicaragua y el Sector Pesquero. Documento actualizado al año 2004. Ministerio De Fomento Industria y Comercio (MIFIC) Administración Nacional de la Pesca (AdPESCA). Managua, Nicaragua. 70 pp.

Guiste H (2005) Dominica shares fish aggregating device technology (FAD) with Grenada in CRFM News: The Biannual Newsletter of the Caribbean Regional Fisheries Mechanism. Issue No. 4, December 2005.

Habteyonas MZ, Scrimgeour F (2003) An economic analysis of artisanal fisheries in Eritrea: identifying the constraints. 2003 New Zealand Association of Economists Conference, Auckland, New Zealand. Available at: http://www.nzae.org.nz/conferences/2003/34-SCRIMGEOUR-report.DOC. Accessed on April 9, 2006.

Hawkins JP, Roberts CM (2004) Effects of artisanal fishing on Caribbean coral reefs. Conserv Biol 18(1): 215-226.

Hawkins JP, Roberts CM, Gell FR, Dytham C (2007) Effects of trap fishing on reef fish communities. Aquat Conserv 17: 111-132.

Hoetjes P, Lum Kong A, Juman R, Miller A, Miller M, et al. (2002) Status of coral reefs in the eastern Caribbean: The ECS, Trinidad and Tobago, Barbados and the Netherlands Antilles; pp. 325-342 in C. Wilkinson, ed. Status of coral reefs of the world. Townsville: Australian Institute of Marine Science.

http://www.accessgambia.com/information/fisheries-sector

http://www.fao.org/DOCREP/003/R0395E/R0395E02.htm

http://www.fao.org/DOCREP/003/R0395E06.htm#fig1

http://www.fao.org/docrep/006/X3125E/X3125E00.HTM.

http://www.illegal-fishing.info/

http://www.persga.org/

INFOPECHE (1999) Liberia - general economic data. 4 pp.

Instituto Nacional de la Pesca (2007a) Resultado del análisis de las poblaciones de camarón del litoral del Pacífico para implementar la veda durante el 2007. Informe Definitivo, pp. 3-4. Secretaría de Medio Ambiente, Recursos Naturales y Pesca, México.

Instituto Nacional de la Pesca (2007b) Resultados de los muestreos de las poblaciones de camarón, durante la veda del 2007 en el litoral del pacífico. Informe Definitivo, pp. 25-26. Secretaría de Medio Ambiente, Recursos Naturales y Pesca, México.

IOTC (2003) National report of South Africa. IOTC -SC-03-Inf.5. 4 pp.

Joyce IT (1997) The Spiny-Lobster Fishery in Cuba. Geogr Rev 87(4): 484-503.

Konan J (1993) The artisanal sardinella fishery in Cote d'Ivoire. Centre de Recherches Océanologiques. Abidjan, Cote d'Ivoire. 11 pp.

Koranteng KA (1992) The Ghanaian fishery for sardinellas. Fisheries Department, Research and Utilization Branch, Tema, Ghana. 16 pp.

Laporta M, Sales G, Arias A, Di Paola JL, Giffoni B, et al. (2004) Descripción de las pesquerías industriales que interactúan con tortugas marinas y revisión de las capturas incidentales reportadas para el Océano Atlántico Sur Occidental. II Reunión Sobre la Investigación y Conservación de Tortugas Marinas del Atlántico Sur Occidental - ASO - 30 de septiembre- 1 y 2 de Octubre, 2004 - San Clemente del Tuyú Bs. As. - Argentina.

Leda Fisheries, Ltd (1999) Leda fish: Eritrea. Available at: http://www.ledafish.com/index1.htm. Accessed on: April 11, 2007.

Luckhurst B, Booth S, Zeller D (2003) Brief history of Bermudian fisheries, and catch comparison between national sources and FAO records. Fisheries Centre Research Reports 11(6): 163-169.

MacRae DR, Esteban N (2007) St. Eustatius Marine Park Management Plan. Coastal Zone Management (UK) and St Eustatius National Parks Foundation (STENAPA). 126 pp.

Marcano LA, Alió JJ (2000) Incidental capture of sea turtles by the industrial shrimping fleet off northeastern Venezuela, 293. Proceedings of the Eighteenth International Sea Turtle Symposium. U.S. Dept. of Commerce. NOAA Technical Memorandum NMFS-SEFSC-436, p. 107.

Matos-Caraballo D (2005) Bycatch study of Puerto Rico's marine commercial fisheries. Final grant report. Puerto Rico Department of Natural and Environmental Resources. 11 pp.

Matos-Caraballo D, Posada JM, Luckhurst BE (2006) Fishery-dependent evaluation of a spawning aggregation of tiger grouper (Mycteroperca tigris) at Vieques Island, Puerto Rico. B Mar Sci 79(1): 1-16.

McConney P (2001) Multi-objective management of inshore fisheries in Barbados: A Biodiversity Perspective. In Blue Millenium. IDRC-CRDI UNEP.

McConney P (2003) Grenada case study: the lobster fishery at Sauteurs. Caribbean Coastal Co-management Guidelines Project. Caribbean Conservation Association, Barbados. 65 pp.

McConney P, Baldeo R (2007) Lessons in co-management from beach seine and lobster fisheries in Grenada. Fish Res 87: 77-85.

McPadden C (1989) The Ecuadorian shrimp trawl trawl fishery, 1974-1985. pp. 197-219, In Olsen S, Arriaga L, eds. A sustainable shrimp mariculture industry for Ecuador. Coastal Resources Center, University of Rhode Island. Narragansett, RI.

McVea TA, Kennelly SJ (2005) Proceedings of the 4th International Fisheries Observer Conference - Sydney, Australia, 8 - 11 November 2004. NSW Department of Primary Industries, Cronulla Fisheries Research Centre of Excellence, Cronulla, Australia. ISBN 1 9208 12 20 2. 230 pp.

Ministerio de Agricultura y Ganadería (2006) CENTRO DE DESARROLLO DE LA PESCA Y LA ACUICULTURA, Unidad de Estadística, El Salvador, Estadísticas Pesqueras y Acuícolas, Año 2006, Vol. 33.

Mohammed E, Vasconcellos M, Mackinson S, Fanning P, Heileman S, et al. (2008) Scientific basis for ecosystem-based management in the Lesser Antilles including interactions with marine mammals and other top predators; a trophic model of the Lesser Antilles pelagic ecosystem. Report prepared for the Lesser Antilles Pelagic Ecosystem Project (gcp/rla/140/jpn). Food and Agriculture Organization of The United Nations, Barbados. 184 pp.

Moses BS (1997) Artisanal fisheries of southeastern Nigeria: catch rates, population dynamics, potential yields and resources managements. Ph.D. Dissertation, University of Uyo, Uyo.

Moses BS, Udoidiong OM, Okon AO (2002) A statistical survey of the artisanal fisheries of south-eastern Nigeria and the influence of hydroclimatic factors on catch and resource productivity. Fish Res 57: 267-278.

Peckham SH, Maldonado D, Walli A, Ruiz G, Nichols WJ, et al. (2007) Small-scale fisheries bycatch jeopardizes endangered Pacific loggerhead turtles. PLoS One 2: doi:10.1371/journal.pone.0001041.

Priyono BE (2003) Socioeconomic and bioeconomic analysis of coastal resources in Central and Northern Java, Indonesia. p. 479 - 516. In G. Silvestre, L. Garces, I. Stobutzki, M. Ahmed, R.A. Valmonte-Santos, C. Luna, L. Lachica-Aliño, P. Munro, V. Christensen and D. Pauly (eds.) Assessment, Management and Future Directions for Coastal Fisheries in Asian Countries. WorldFish Center Conference Proceedings 67, 1 120 pp.

Project GloBAL (2006) Project GloBAL country profiles. Duke Center for Marine Conservation, Duke University Marine Laboratory, Beaufort, NC. <http://bycatch.env.duke.edu/>

Project GloBAL (2007) Project GloBAL country profiles. Duke Center for Marine Conservation, Duke University Marine Laboratory, Beaufort, NC. <http://bycatch.env.duke.edu/>

Project GloBAL (2008) Project GloBAL country profiles. Duke Center for Marine Conservation, Duke University Marine Laboratory, Beaufort, NC. <http://bycatch.env.duke.edu/>

Project GloBAL (2009) Project GloBAL country profiles. Duke Center for Marine Conservation, Duke University Marine Laboratory, Beaufort, NC. <http://bycatch.env.duke.edu/>

Reyes JC, Oprto JA (1994) Gillnet fisheries and cetaceans in the southeast Pacific. Report of the International Whaling Commission. Special Issue 15. SC/090/G11:467-474.

Reynolds JE, Appleton J, Bellemans MS, Bozon A, Christy LC (1993) The Fisheries of Eritrea, Part I: Sector Review. Part II: Proposed national fisheries strategy and development programme. FAO/TCP/ERT-2261. Fisheries Department, FAO. Available at: http://www.fao.org/docrep/field/003/ab902e/ab902e00.htm and http://www.fao.org/docrep/field/003/ab902e/AB902E05.htm. Accessed on: December 22, 2006.

Rivera CC, Palacios NA (2005) Anuario Pesquero y Acuícola 2004. Centro de Investigaciones Pesqueras y Acuícolas (CIPA) de la Administración Nacional de Pesca y Acuicultura (AdPesca). Managua, Nicaragua. 51 pp.

Rivera CC, Palacios NA, Brenes BS (2006) Anuario Pesquero y Acuícola 2005. Centro de Investigaciones Pesqueras y Acuícolas (CIPA) de la Administración Nacional de Pesca y Acuicultura (AdPesca). Managua, Nicaragua. 56 pp.

Rudd MA (2003) Fisheries landing and trade of the Turks and Caicos Islands. In From Mexico to Brazil: Central Atlantic fisheries catch trends and ecosystem models. Fisheries Center Research Reports 11(6): pp149-161. University British Columbia, Vancouver, Canada. 264 pp.

Sadovy Y, Eklund AM (1999) Synopsis of biological data on the Nassau grouper, *Epinephelus* *striatus* (Bloch 1792), and the Jewfish, *E. itajara* (Lichtenstein, 1822). NOAA Technical Report 146. A Technical Report of the Fishery Bulletin. FAO Fisheries Synopsis 157. U.S. Department of Commerce. Seattle, Washington. 68 pp.

SAGARPA 1 (2004) Diario Oficial. Segunda Sección. Acuerdo mediante el cual se aprueba la actualización de la Carta Nacional Pesquera y su anexo. 112 pp.

Salas SR, Chuenpagdee R, Seijo JC, Charles A (2007) Challenges in the assessment and management of small-scale fisheries in Latin America and the Caribbean. Fish Res 87: 5-16.

Samba A, Samb B (1993) Senegalese canoe fishery for sardinella. Chercheurs au Centre de Recherches Océanographiques de Dakar Thiaroye, C.R.O.D.T/ISRA - Sénégal. 16 pp.

Sancho Andrade A, Ortiz-von Halle B, Naranjo Cuvi N (2002) La pesca y el comercio de bacalao de profundidad Dissostichus eleginoides en América del Sur: una perspectiva regional. Un reporte de TRAFFIC América del Sur. 191 pp.

Santana Hernández H, Valdez Flores JJ (2006) Selectividad y eficiencia del palangre de deriva con tres tipos de anzuelo y dos tipos de carnada en la pesca de tiburón. Technical Report. SAGARPA, Instituto Nacional de la Pesca. Manzanillo, Colima, Mexico. 14 pp.

Sebastian RD (2002) National report of the Commonwealth of Dominica. Pp. 27-34 in FAO Fisheries Report No. 683, Supplement. Western Central Atlantic Fishery Commission: National reports and technical papers presented at the first meeting of the WECAFC Ad Hoc Working Group on the development of sustainable moored fish aggregating device fishing in the Lesser Antilles. Le Robert, Martinique, 8-11 October 2001. Food and Agriculture Organization of the United Nations. Rome.

SEDAR. SEDAR 14 Caribbean Yellowfin Grouper, Mutton Snapper, Queen Conch. http://www.sefsc.noaa.gov/sedar/Sedar_Documents.jsp?WorkshopNum=14&FolderType=Assessment

Segura Valle JH (1999) Censo de embarcaciones pesqueras artesanales y de pequeña escala. Ministerio de Agricultura Ganaderia y Alimentacion Unidad de Manejo de la Pesca y la Acuicultura (UNIPESCA), Programa Regional de Apoyo al Desarrollo de la Pesca Artesanal en el Istmo Centroamericano (PRADEPESCA), Ministerio de la Defensa Nacional, Base Naval del Pacifico, Base Naval del Atlantico, Federacion Nacional de Pescadores Artesanales de Guatemala (FENAPESCA). Informe Nacional, Guatemala. 53 pp.

SEMARNAT (2005) Captílulo 5. Aprovechamiento de los recursos forestales, pesqueros y de la vida silvestre.

Suman D (2006) The case of the shrimp industry in eastern Panama (Darién Province): unsustainable harvest of a valuable export product and its limited impact on local community development; pp. 192-220. In RL Harris (ed.). Globalization and Sustainable Development: Issues and Applications. Patel Center for Global Solutions: University of South Florida.

ter Hofstede R, Dickey-Collas M (2006) An investigation of seasonal and annual catches and discards of the Dutch pelagic freezer-trawlers in Mauritania, Northwest Africa. Fish Res 77: 184-191.

Trott T, Luckhurst BE, Medley P (2002) Report on the spiny lobster fishery of Bermuda. Report of the second workshop on the management of Caribbean spiny lobster fisheries in the WECAFC area. FAO Fisheries Report No. 715. Part 2.3. Food and Agriculture Organization of the United Nations. Rome.

Tsehaye I, Machiels MAM, Nagelkerke LAJ (2007) Rapid shifts in catch composition in the artisanal Red Sea reef fisheries of Eritrea. Fish Res 86: 58-68.

Tudela S, Kai Kai A, Maynou F, El Andalossi M, Guglielmi P (2005) Driftnet fishing and biodiversity conservation: the case study of the large-scale Moroccan driftnet fleet operating in the Alboran Sea (SW Mediterranean). Biol Conserv 121: 65-78.

Ulloa JB, Salazar JL, Pacheco R, Portillo CJ, Moreno JF, et al. (2003) Monitoreo y evaluación del recurso camarón de mar en El Salvador (período de Julio de 1998 a Octubre de 1999). Ministerio de Agricultura y Ganaderia Centro de Desarrollo de la Pesca y la Acuicultura Unidad de Pesquerías. Santa Tecla, El Salvador. 22 pp.

UNIPESCA (2003) Boletín Estadístico de la Pesca y Agricultura Períodio 1991-2001. Ministerio de Agricultura, Ganadería y Alimentación, Unidad de Manejo de la Pesca y Acuicultura. Guatemala, Centro América. 81 pp.

UNIPESCA (2004) Censo de pesca artesanal y pequeña escala que opera en aguas marino costeras de Guatemala. Proyecto apoyo a la pesca artesanal en Guatemala Unidad de Manejo de la Pesca y Acuicultura (UNIPESCA), Agencia Española de Cooperación Internacional (AECI). 34 pp.

Ustate Duarte EZ (2002) Diagnóstico de la cadena productiva pesquera en la República de Colombia. Estudio de prospectiva para la cadena productiva de la industria pesquera en la región de la costa del Pacífico en América del Sur. Project No.: US/RLA/02/149. 29 pp.

Weidner DM, Serrano JA (1997) World Swordfish Fisheries, Volume IV: Latin America, Part A. South America, Section 1. Pacific, Segment A. Colombia, Ecuador, and Peru. NOAA Tech. Memo. NMFS-F/SPO-261997: 151-273.

Weidner DM, Laya GE, Folsom WB (2001) An analysis of swordfish fisheries, market trends, and trade patterns, past-present-future. Volume IV: Latin America, Part B: Caribbean, Section 3: Dominica to Martinique. Prepared by The Office of Science and Technology.

Yearbook of Statistics Sabah (2005) Department of Statistics. Malaysia, Sabah.

Zeeberg JJ, Corten AHM, de Graaf E (2006) Bycatch and release of pelagic megafauna in industrial fisheries off Northwest Africa. Fish Res 78: 185-196.
